# Case Report: Primary hepatic carcinosarcoma with ectopic β-hCG secretion and a paraneoplastic leukemoid reaction mimicking gynecologic and hematologic disease

**DOI:** 10.3389/fonc.2026.1864613

**Published:** 2026-06-24

**Authors:** Pin Wang, Xin Xu, Lan Zhang

**Affiliations:** 1 Department of Hepatobiliary Oncology, Zhongshan Hospital, Fudan University, Shanghai, China; 2 Liver Cancer Institute, Zhongshan Hospital, Key Laboratory of Carcinogenesis and Cancer Invasion (Ministry of Education), Fudan University, Shanghai, China

**Keywords:** diagnostic pitfall, ectopic β-hCG secretion, leukemoid reaction, liver tumor, paraneoplastic syndrome, primary hepatic carcinosarcoma

## Abstract

Primary hepatic carcinosarcoma is an exceptionally rare and highly aggressive malignancy, and ectopic β-hCG production and paraneoplastic leukemoid reaction are both uncommon findings in solid tumors. We report the case of a 47-year-old woman who initially presented with abnormal vaginal bleeding and elevated serum β-hCG, raising concern for a pregnancy-related disorder. Diagnostic curettage showed no evidence of pregnancy or trophoblastic disease, yet the β-hCG level continued to rise. At the same time, she developed extreme leukocytosis, and bone marrow examination favored a leukemoid reaction rather than hematologic malignancy. Imaging subsequently revealed a rapidly enlarging mass in the right hepatic lobe with necrosis, satellite lesions, vascular involvement, and hilar nodal disease. Surgical resection was performed because the lesion was considered resectable and tissue diagnosis was required. Histopathology demonstrated a poorly differentiated primary hepatic carcinosarcoma with β-hCG expression in a subset of tumor cells. After surgery, the white blood cell count, serum β-hCG, and PIVKA-II levels all fell markedly, supporting their paraneoplastic origin. However, the tumor recurred rapidly, and the patient died approximately 1 month after surgery. This case shows that persistent β-hCG elevation and marked leukocytosis, when unexplained by gynecologic or hematologic disease, may rarely be the presenting clues to an aggressive primary hepatic malignancy. Importantly, persistent β-hCG elevation and marked leukocytosis should not be interpreted in isolation as gynecologic or hematologic disease when imaging reveals a rapidly enlarging liver mass.

## 1 Introduction

Primary hepatic carcinosarcoma is a very rare malignant tumor of the liver with highly aggressive behavior and poor prognosis (1, 2, 5). It is characterized by the coexistence of carcinomatous and sarcomatous elements and is often associated with rapid growth, extensive necrosis, vascular invasion, early metastasis, and short survival (1–3, 5). Because both the clinical presentation and imaging findings are usually nonspecific, preoperative diagnosis is difficult, and the final diagnosis often depends on histopathologic and immunohistochemical evaluation (2, 4, 5).

Ectopic β-human chorionic gonadotropin (β-hCG) production in nongestational tumors is uncommon, and reports involving primary hepatic malignancies are particularly rare (6). In parallel, a paraneoplastic leukemoid reaction is also an unusual manifestation of solid tumors and may closely mimic a primary hematologic disorder (10, 11), especially when accompanied by marked leukocytosis and diffuse bone marrow hypermetabolism on positron emission tomography/computed tomography (7–9). When these two findings occur together, the diagnostic workup may be diverted toward gynecologic or hematologic disease, delaying recognition of the underlying malignancy.

Here, we report a case of primary hepatic carcinosarcoma in a 47-year-old woman who initially presented with abnormal vaginal bleeding, persistently elevated β-hCG, and extreme leukocytosis. The case was diagnostically challenging because it created simultaneous suspicion for a pregnancy-related disorder and a hematologic malignancy. The postoperative fall in β-hCG, white blood cell count, and PIVKA-II strongly supported their paraneoplastic relationship to the hepatic tumor. This case highlights an unusual presentation of an aggressive primary hepatic malignancy and underscores the importance of considering a solid tumor when elevated β-hCG and marked leukocytosis cannot be explained by gynecologic or hematologic evaluation.

## 2 Case report

A 47-year-old woman initially presented to a local hospital with increased vaginal bleeding during menstruation. Her serum β-human chorionic gonadotropin (β-hCG) level was 162.9 mIU/mL, and a pregnancy-related disorder was first suspected. She underwent diagnostic curettage, but histopathologic examination showed only an endometrial polyp and simple endometrial hyperplasia, without evidence of pregnancy or trophoblastic disease. After the procedure, the β-hCG level did not fall and instead continued to rise, fluctuating between 300 and 700 mIU/mL. During the same period, she developed marked leukocytosis, with a peak white blood cell count of 97.05 × 10^9^/L. Bone marrow aspiration performed at the referring hospital favored a leukemoid reaction rather than a primary hematologic malignancy.

Abdominal ultrasonography subsequently detected a mass in the right anterior liver measuring approximately 92 × 86 mm. Repeat ultrasonography 18 days later showed rapid enlargement to 120 × 112 × 143 mm. Concurrent gynecologic ultrasonography showed no definite evidence of intrauterine or ectopic pregnancy. Contrast-enhanced abdominal magnetic resonance imaging demonstrated a large mass in the right hepatic lobe, measuring approximately 121 × 133 × 128 mm, with heterogeneous peripheral enhancement in the arterial phase and progressive peripheral enhancement on delayed-phase imaging. The patient reported right upper abdominal fullness, but no fever, jaundice, nausea, vomiting, abdominal pain, or diarrhea. There was no fever or other clear clinical evidence of active infection to account for the extreme leukocytosis. Her medical history was notable for chronic hepatitis B virus infection for 30 years without antiviral treatment.

**Figure 1:**
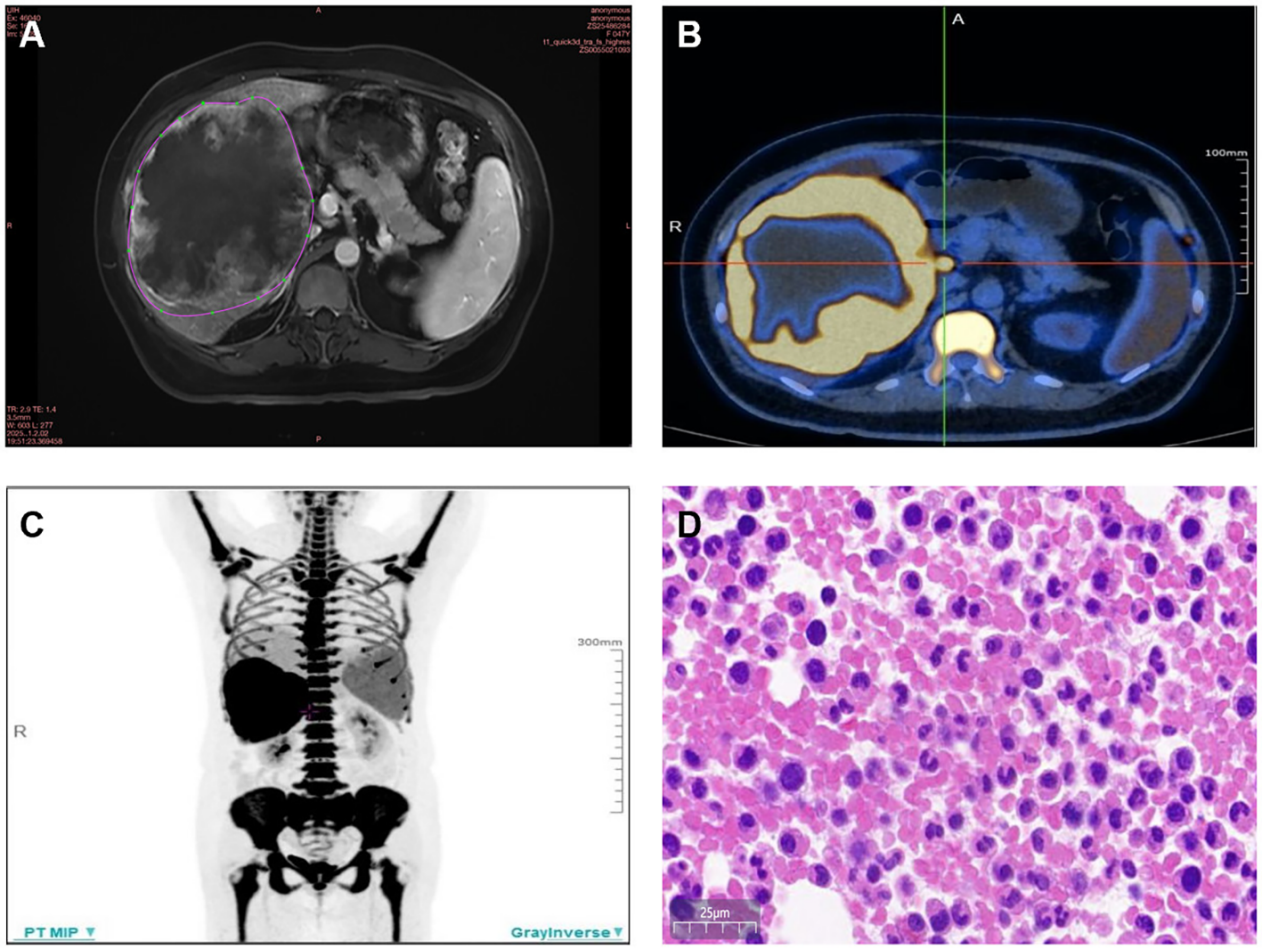
Diagnostic workup showing the hepatic lesion and the misleading hematologic findings. **(A)** Contrast-enhanced abdominal magnetic resonance imaging shows a large heterogeneous mass in the right anterior liver, with internal necrosis and involvement of adjacent hepatic vessels. **(B)** ^18F-FDG PET/CT fusion imaging demonstrates intense hypermetabolism of the hepatic mass with a central photopenic necrotic area. **(C)** Maximum intensity projection image from ^18F-FDG PET/CT shows diffuse bone marrow hypermetabolism and increased splenic uptake, initially raising concern for a hematologic malignancy. **(D)** Bone marrow aspirate smear shows marked granulocytic hyperplasia with left shift and no excess blasts, favoring a leukemoid reaction rather than a primary hematologic malignancy.

She was then admitted to our hospital for further evaluation. Repeat transvaginal ultrasonography again showed no definite intrauterine or ectopic pregnancy, although uterine fibroids and bilateral adnexal cystic or mixed echogenic lesions were noted. Contrast-enhanced abdominal MRI confirmed a massive tumor in the right anterior liver with restricted diffusion, heterogeneous enhancement, internal necrosis, surrounding satellite lesions, and involvement of adjacent hepatic veins and portal vein branches (**Figure 1A**). During hospitalization, the white blood cell count remained markedly elevated at 55.37–80.34 × 10^9^/L, and the β-hCG level further increased to 963.0–1185 mIU/mL. Prothrombin induced by vitamin K absence-II (PIVKA-II) was markedly elevated at 1090 mAU/mL, whereas alpha-fetoprotein was 1.5 ng/mL. Several inflammatory cytokines were also elevated.

To further assess the extent of disease, ^18F-fluorodeoxyglucose positron emission tomography/computed tomography (^18F-FDG PET/CT) was performed. It showed a large hypermetabolic mass in the right hepatic lobe extending into the left medial lobe, with central photopenia consistent with necrosis, together with hypermetabolic hilar lymph nodes (**Figure 1B**). The spleen was enlarged and diffusely hypermetabolic, and the maximum intensity projection image showed diffuse bone marrow hypermetabolism throughout the body (**Figure 1C**). Pelvic lesions involving the uterus and adnexa showed only low metabolic activity. Because of the diffuse marrow uptake, a second bone marrow aspiration was performed. This again showed no evidence of hematologic malignancy, but instead demonstrated marked granulocytic hyperplasia with relative erythroid suppression, consistent with a leukemoid reaction (**Figure 1D**). Thus, the negative gynecologic and hematologic workups, together with the rapidly enlarging hepatic mass and PET/CT findings, redirected the diagnostic focus toward an aggressive hepatic malignancy.

**Figure 2:**
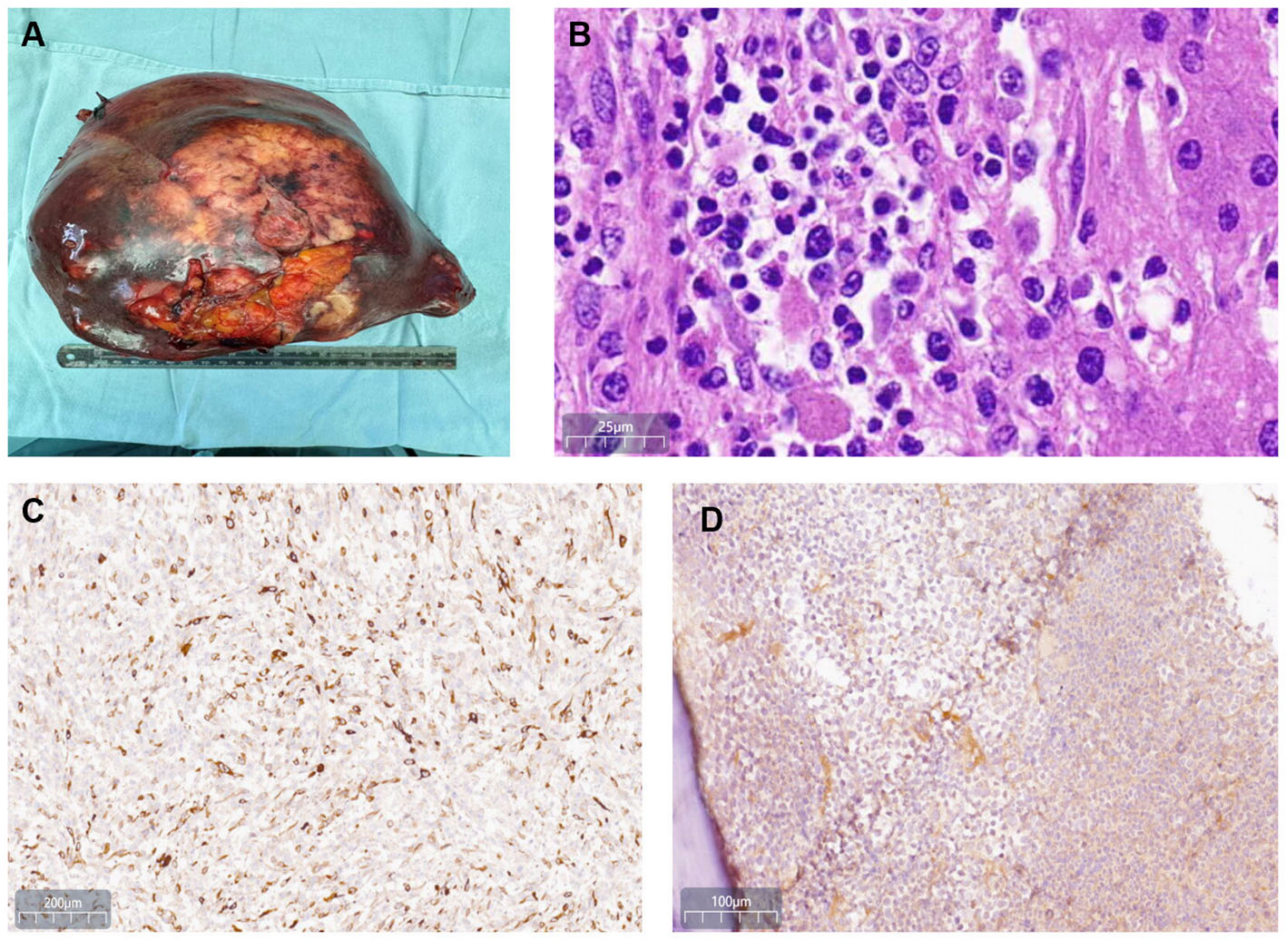
Pathologic findings supporting primary hepatic carcinosarcoma with β-hCG expression. **(A)** Gross specimen of the resected hepatic tumor shows a large mass with extensive hemorrhage and necrosis. **(B)** Hematoxylin and eosin staining shows a poorly differentiated malignant neoplasm composed of epithelioid and spindle-cell elements with marked pleomorphism and brisk mitotic activity. **(C)** Immunohistochemical staining for β-hCG shows cytoplasmic positivity in a subset of tumor cells, supporting ectopic β-hCG production by the tumor. **(D)** Bone marrow specimen shows β-hCG immunoreactivity without morphologic evidence of metastatic tumor involvement.

After multidisciplinary discussion involving hematology, gynecology, nuclear medicine, radiology, and hepatobiliary surgery, the liver lesion was considered highly likely to be malignant. Percutaneous biopsy was thought to carry a substantial risk of bleeding, and the tumor appeared resectable. Surgical resection was therefore selected because it could provide both definitive diagnosis and local tumor control. The patient therefore underwent laparoscopic abdominopelvic exploration with resection of the right ovarian lesion, followed by open partial hepatectomy involving segments V, VI, VII, and VIII, together with cholecystectomy and radiofrequency ablation of a separate hepatic lesion. Intraoperatively, a large necrotic hepatic tumor was identified. Gross examination showed a 17.5 × 15.0 × 12.0 cm mass with a variegated cut surface and extensive hemorrhage and necrosis (**Figure 2A**).

Histopathologic examination showed a poorly differentiated malignant hepatic neoplasm composed of epithelioid and spindle cells with marked pleomorphism, tumor giant cells, extensive tumor necrosis, and brisk mitotic activity (**Figure 2B**). Numerous vascular tumor emboli were present, consistent with M2a high-risk microvascular invasion, and the tumor also invaded the liver capsule. Immunohistochemistry did not support a specific line of differentiation. However, the morphologic coexistence of epithelioid and spindle-cell/sarcomatoid malignant components, together with marked pleomorphism, extensive necrosis, brisk mitotic activity, and vascular invasion, supported the diagnosis of carcinosarcoma. Only a small subset of tumor cells showed pan-cytokeratin positivity, whereas HCG was positive in approximately 20% of cells, WT-1 in approximately 70%, and SMAD4 was retained. AFP, Hepa, and GPC3 were all negative. β-hCG immunostaining showed cytoplasmic positivity in a subset of tumor cells (**Figure 2C**). Bone marrow tissue also showed β-hCG immunoreactivity, although no morphologic evidence of metastatic tumor involvement was identified (**Figure 2D**). Taken together with the morphologic findings and the clinical course, these results supported the diagnosis of primary hepatic carcinosarcoma with ectopic β-hCG secretion and a paraneoplastic leukemoid reaction. Pathologic examination of a bowel wall nodule and a perirenal fat nodule also showed metastatic poorly differentiated malignant tumor. The resected right ovarian lesion was benign and favored an inclusion cyst. Together with the negative gynecologic evaluations and the absence of trophoblastic disease on diagnostic curettage, the benign ovarian pathology made gynecologic malignancy, trophoblastic disease, germ cell tumor, and metastatic tumors less likely.

After surgery, the white blood cell count fell rapidly to 15.62–22.80 × 10^9^/L, the β-hCG level decreased to 54.3–67.1 mIU/mL, and PIVKA-II dropped from 1090 mAU/mL to 18 mAU/mL. Liver function tests rose transiently after surgery and then improved. She recovered uneventfully in the early postoperative period and was discharged 2 weeks after the operation. The major clinical events and dynamic changes in key laboratory parameters are summarized in **Table 1**.

**Table 1:** Timeline of major clinical events and dynamic changes in key laboratory parameters.

Clinical stage/time point	Major clinical events	White blood cell count (3.5–9.5 × 10^9^/L)	Serum β-hCG (mIU/mL)	PIVKA-II (0–40 mAU/mL)	Serum calcium (2.11–2.52 mmol/L)	Interleukin-6 (0–7 pg/mL)	Interleukin-8 (0–62 pg/mL)
Preoperative phase	Abnormal bleeding; rising β-hCG; marked leukocytosis; liver mass detected	80.34	1185	1090	2.31	274.4	109.5
Early postoperative phase	Partial hepatectomy; transient postoperative improvement	15.62	54.3	18	—	—	—
Recurrence phase	Early recurrence with systemic deterioration	77.17	—	64	3.37	94.3	1347.6

β-hCG, beta-human chorionic gonadotropin; PIVKA-II, prothrombin induced by vitamin K absence-II. **—** indicates that the value was not measured at that time point.

**Figure 3:**
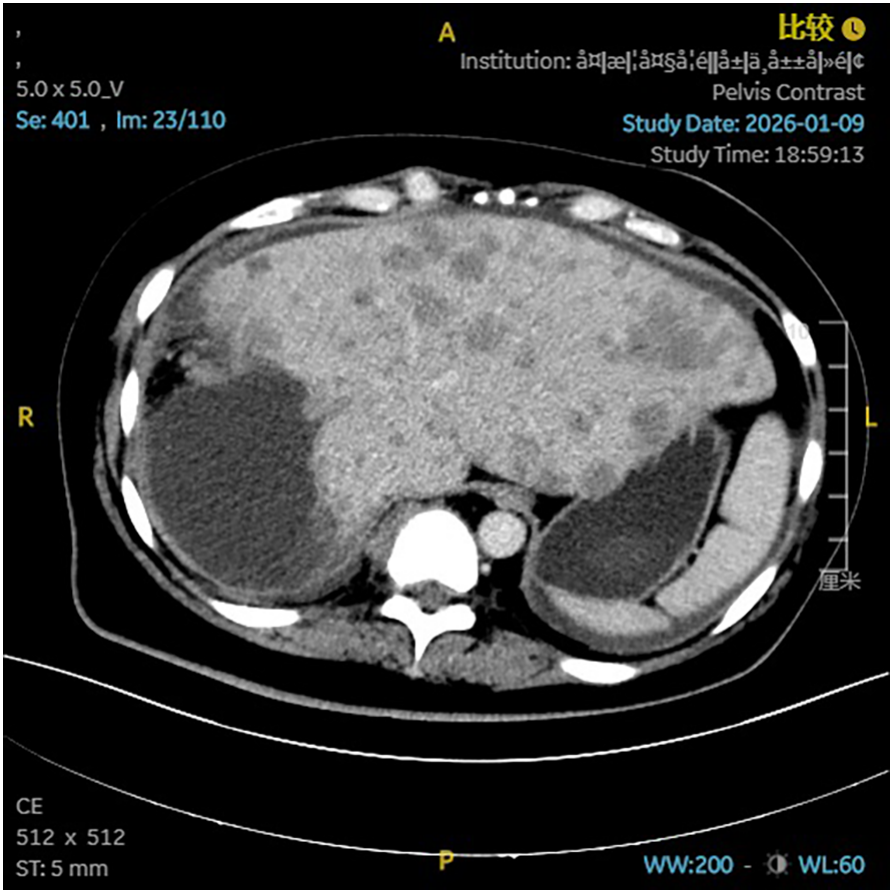
Postoperative computed tomography showing early recurrence in the remnant liver. Contrast-enhanced abdominal computed tomography obtained approximately 1 month after surgery shows multiple recurrent metastatic lesions throughout the remnant liver, consistent with rapid postoperative progression.

Her condition, however, worsened quickly. She first developed shoulder pain that did not improve with routine treatment at a local hospital, followed by abdominal distension, bilateral lower-extremity edema, numbness of the fingers, and weakness of the upper limbs. Approximately 1 month after surgery, she was readmitted with extreme leukocytosis, hypercalcemia, recurrent elevation of PIVKA-II, cytokine storm-like changes, and marked deterioration in liver function. Contrast-enhanced abdominal computed tomography showed multiple recurrent metastatic lesions throughout the remnant liver, consistent with rapid postoperative progression (**Figure 3**). Despite intensive supportive care, her condition deteriorated irreversibly, and she died approximately 1 month after the operation.

## 3 Discussion

Primary hepatic carcinosarcoma is an exceptionally rare liver malignancy composed of both carcinomatous and sarcomatous elements. Because reported cases remain limited, its clinicopathologic spectrum is still not well defined, and most clinicians have very little direct experience with it. What is clear from the published literature is that this tumor usually behaves in a highly aggressive manner, often presenting as a large mass with necrosis, vascular invasion, rapid progression, and early metastasis (1–5). Our patient followed that pattern closely: the tumor enlarged quickly over a short interval, showed extensive necrosis and vascular involvement on imaging, and recurred explosively soon after surgery.

The most unusual aspect of this case was not only the rarity of the tumor itself, but the combination of two misleading paraneoplastic findings. To the best of our knowledge, this combination has not been previously reported in primary hepatic carcinosarcoma. That point matters because each of these abnormalities can redirect the diagnostic workup on its own. Elevated β-hCG naturally raises concern for pregnancy-related disease or trophoblastic neoplasia, whereas extreme leukocytosis with diffuse marrow uptake on PET/CT strongly suggests a hematologic disorder. When both appear together, the possibility of an aggressive primary hepatic malignancy may be overlooked at the beginning. Published evidence on β-hCG-producing primary liver tumors and leukemoid reaction in sarcomatoid or carcinosarcomatous hepatic malignancies remains limited.

Persistent β-hCG elevation was one of the earliest clues in this patient, but it was also one of the most deceptive. Ectopic hCG production by non-gestational tumors is well recognized, yet reports involving primary liver tumors are uncommon. An older immunohistochemical study showed that hCG can be detected in a small subset of poorly differentiated primary liver carcinoma cells, which makes the biology of our case plausible, but published evidence remains very limited (6). In our patient, repeated gynecologic evaluation did not identify an intrauterine or ectopic source, while the hepatic tumor showed β-hCG expression and the serum β-hCG level fell sharply after resection. Taken together, these findings strongly support the liver tumor as the source of ectopic β-hCG. These findings also helped distinguish the condition from pregnancy-related disease, trophoblastic disease, germ cell tumor, or gynecologic malignancy in the clinical differential diagnosis.

The leukemoid reaction created a second major diagnostic trap. There is precedent for white blood cell elevation in rare hepatic sarcomatoid malignancies: a prior case of carcinosarcoma of the liver was reported to produce granulocyte colony-stimulating factor (7), and paraneoplastic leukemoid reaction has also been described in sarcomatoid hepatocellular carcinoma (8, 9). Still, that is not the same as the combination seen here (10, 11). In this patient, the white blood cell count rose to an extreme level, PET/CT showed diffuse marrow hypermetabolism, and the overall picture strongly resembled a hematologic neoplasm. However, two bone marrow examinations failed to show leukemia or another primary blood malignancy and instead supported reactive granulocytic hyperplasia. This mismatch between dramatic hematologic findings and nondiagnostic marrow pathology was a key clue that the leukocytosis was paraneoplastic rather than primary. The absence of fever or other clear clinical evidence of active infection further supported that infection was unlikely to be the main cause of the marked leukocytosis. This interpretation was therefore more consistent with a tumor-related paraneoplastic leukemoid reaction than with leukemia or another primary hematologic malignancy.

Another important feature of this case was the dynamic behavior of the laboratory markers. After surgery, the white blood cell count, serum β-hCG, and PIVKA-II all dropped rapidly. When the disease recurred, the clinical condition worsened just as quickly, with recurrent leukocytosis, rising tumor-related abnormalities, and widespread progression on imaging. In a case with several competing diagnostic explanations, this parallel rise-and-fall pattern helped unify the picture. This temporal pattern supports an association between these marker changes and tumor burden. Because direct mechanistic assays were not performed, this relationship should be interpreted as supportive rather than definitive evidence of causality.

From a practical standpoint, this case carries a simple but important message. When persistent β-hCG elevation cannot be explained by gynecologic disease (6), and marked leukocytosis is not supported by bone marrow evidence of hematologic malignancy (10, 11), clinicians should widen the differential diagnosis rather than pursue each abnormality in isolation. If imaging at the same time reveals a rapidly enlarging hepatic mass, an aggressive primary liver tumor should be considered, even when the initial presentation seems to point elsewhere. In that sense, the value of this case lies not only in its rarity, but also in the diagnostic lesson it offers. In practical terms, unexplained β-hCG elevation and leukemoid reaction after standard gynecologic and hematologic evaluation should prompt clinicians to broaden the differential diagnosis and actively search for an underlying solid tumor.

Several limitations should be acknowledged. This is a single case report, molecular profiling was not available, and serum or tissue G-CSF/GM-CSF was not tested. Therefore, the biological mechanisms underlying β-hCG production and the leukemoid reaction could not be directly proven. The paraneoplastic interpretation is therefore supported mainly by the clinical course, immunohistochemical findings, and dynamic postoperative laboratory changes rather than by direct functional assays.

## 4 Conclusion

Primary hepatic carcinosarcoma is an exceptionally rare and highly aggressive malignancy (1–5). In this case, the tumor presented with two particularly misleading findings, persistent β-hCG elevation and a leukemoid reaction, which initially suggested gynecologic and hematologic disease rather than a primary liver tumor. To our knowledge, this combination has not been previously reported in primary hepatic carcinosarcoma. This case shows that when elevated β-hCG and marked leukocytosis cannot be explained by routine gynecologic or hematologic evaluation, an aggressive solid tumor should also be considered. Integrating imaging, pathology, and dynamic laboratory changes is essential for reaching the correct diagnosis in such complex presentations.

## Data Availability

The original contributions presented in the study are included in the article/supplementary material. Further inquiries can be directed to the corresponding author.

## References

[1] Lao XM Chen DY Zhang YQ Xiang J Guo RP Lin XJ . Primary carcinosarcoma of the liver: clinicopathologic features of 5 cases and a review of the literature. Am J Surg Pathol. (2007) 31:817–26. doi: 10.1097/01.pas.0000213431.07116.e0 17527068

[2] Wang QB Cui BK Weng JM Wu QL Qiu JL Lin XJ . Clinicopathological characteristics and outcome of primary sarcomatoid carcinoma and carcinosarcoma of the liver. J Gastrointest Surg. (2012) 16:1715–26. doi: 10.1007/s11605-012-1946-y 22767081

[3] Lin YS Wang TY Lin JC Wang HY Chou KF Shih SC . Hepatic carcinosarcoma: clinicopathologic features and a review of the literature. Ann Hepatol. (2013) 12:495–500. doi: 10.1016/s1665-2681(19)31015-4 23619269

[4] Li J Liang P Zhang D Liu J Zhang H Qu J . Primary carcinosarcoma of the liver: imaging features and clinical findings in six cases and a review of the literature. Cancer Imaging. (2018) 18:7. doi: 10.1186/s40644-018-0141-0 29482629 PMC5828419

[5] Bin F Chen Z Liu P Liu J Mao Z . The clinicopathological and imaging characteristics of primary hepatic carcinosarcoma and a review of the literature. J Hepatocell Carcinoma. (2020) 7:169–80. doi: 10.2147/jhc.s272768 33117751 PMC7568615

[6] Nakanuma Y Sasaki M Yamasaki S Harada K . Human chorionic gonadotropin in primary liver carcinoma in adults. An immunohistochemical study. Virchows Arch A Pathol Anat Histopathol. (1986) 409:365–73. doi: 10.1007/bf00708253 3014718

[7] Aita K Seki K . Carcinosarcoma of the liver producing granulocyte-colony stimulating factor. Pathol Int. (2006) 56:413–9. doi: 10.1111/j.1440-1827.2006.01979.x 16792552

[8] Shin HP Jeon JW Lee SH Choi D Jang KT Kim YS . A case of leukemoid reaction in a patient with sarcomatous hepatocellular carcinoma. Korean J Hepatol. (2011) 17:226–8. doi: 10.3350/kjhep.2011.17.3.226 22102390 PMC3304658

[9] Hu B Sang XT Yang XB . Paraneoplastic leukemoid reaction in a patient with sarcomatoid hepatocellular carcinoma: A case report. World J Clin cases. (2019) 7:1330–6. doi: 10.12998/wjcc.v7.i11.1330 31236397 PMC6580346

[10] Chakraborty S Keenportz B Woodward S Anderson J Colan D . Paraneoplastic leukemoid reaction in solid tumors. Am J Clin Oncol. (2015) 38:326–30. doi: 10.1097/coc.0b013e3182a530dd 24145395

[11] Abukhiran I Mott SL Bellizzi AM Boukhar SA . Paraneoplastic leukemoid reaction: Case report and review of the literature. Pathol Res Pract. (2021) 217:153295. doi: 10.1016/j.prp.2020.153295 33341546

